# Global Sensitivity Analysis of Factors Influencing the Surface Temperature of Mold during Autoclave Processing

**DOI:** 10.3390/polym16050705

**Published:** 2024-03-05

**Authors:** Jiayang He, Lihua Zhan, Youliang Yang, Yongqian Xu

**Affiliations:** 1 Light Alloys Research Institute, Central South University, Changsha 410083, China; jiayanghe_2006@126.com; 2 Department of Energy and Electrical Engineering, Hunan University of Humanities, Science and Technology, Loudi 417000, China; 3 College of Mechanical and Electrical Engineering, Central South University, Changsha 410083, China; yongqian.xu@csu.edu.cn

**Keywords:** autoclave processing, CFRP, mold surface temperature, sensitivity analysis, Sobol method, mold design

## Abstract

During the process of forming carbon fiber reinforced plastics (CFRP) in an autoclave, deeply understanding the global sensitivity of factors influencing mold surface temperature is of paramount importance for optimizing large frame-type mold thermally and enhancing curing quality. In this study, the convective heat transfer coefficient (CHTC), the thickness of composite laminates (TCL), the thickness of mold facesheet (TMF), the mold material type (MMT), and the thickness of the auxiliary materials layer (TAL) have been quantitatively assessed for the effects on the mold surface temperature. This assessment was conducted by building the thermal–chemical curing model of composite laminates and utilizing the Sobol global sensitivity analysis (GSA) method. Additionally, the interactions among these factors were investigated to gain a comprehensive understanding of their combined effects. The results show that the sensitivity order of these factors is as follows: CHTC > MMT > TMF > TCL > TAL. Moreover, CHTC, MMT, and TMF are the main factors influencing mold surface temperature, as the sum of their first-order sensitivity indices accounts for over 97.3%. The influence of a single factor is more significant than that of the interaction between factors since the sum of the first-order sensitivity indices of the factors is more than 78.1%. This study will support the development of science-based guidelines for the thermal design of molds and associated heating equipment design.

## 1. Introduction

Autoclave processing is widely used in the aerospace industry to manufacture composite components due to its ability to ensure high quality, high efficiency, and large-scale production. During autoclave processing, the temperature distribution of composite components is closely tied to that of the mold because of the prepreg lay-up on the mold facesheet. Moreover, the thermal capacity of the mold exceeds that of the composite components, a ratio that increases with the enlargement of molds. As a result, in larger molds, the temperature distribution of the composite part mainly depends on the mold’s temperature field during autoclave processing. Poorly designed molds can cause excessive temperature gradients in the composite, leading to uneven curing, shrinkage, and thermal strains. This, in turn, produces residual stresses and undermines product quality and mechanical performance [1,2,3]. Therefore, the thermal behavior of the mold significantly affects the quality of autoclaved composite components [4], highlighting the importance of thoughtful thermal design in improving composite part quality. In recent years, efforts have been made in the optimization of mold to reduce the temperature gradient and promote thermal performance.

To improve the uniformity of temperature in the mold, research studies have been performed on adjusting the geometric features of the mold, such as the facesheet thickness and substructure construction, and the mold material selection. For example, Zhao et al. [5] proposed a method to modify the facesheet thickness, i.e., increasing it in overheated regions and decreasing it in underheated regions. This approach was validated through computational fluid dynamics (CFD) modeling. The results demonstrated that by adopting this uneven thickness design, the temperature of the mold facesheet is more uniform.

Based on the numerical simulation and genetic algorithm, Wang et al. [6] optimized the substructure construction of the mold, such as the number of grid-boards, thickness of grid-boards, shape and area of the ventilation holes. The results show that the design of the substructure is crucial to the thermal performance of the mold, and the curing synchronization of the composite components was improved by 17.21% after optimization. Furthermore, they utilized a local insulation substructure under the mold facesheet to alter the local CHTC in that area. This alteration decreases the heat flux in overheated areas of the mold facesheet, enhancing the evenness of temperature distribution. By optimizing the shape and layout of the insulation substructure, the maximum temperature difference within C-beam composite components was reduced by 45.69% [7]. Based on the inspiration from the swimming of manta rays, Li et al. [8] proposed a mold with a bionic substructure that can accelerate airflow velocity inside the substructure, and reduce maximum temperature difference and low-temperature areas of mold facesheet. This biomimetic substructure improves the heating efficiency and temperature uniformity of the mold. Yue et al. [9] suggested a topology optimization method for mold substructures. The results showed that the temperature uniformity of the optimized mold increased by 29%, and the curing synchronization of the composite components improved by 26.7%. The above-mentioned studies achieved significant improvements in the heating efficiency and uniformity of the mold by optimizing the mold substructure, increasing the airflow velocity and CHTC near the underheat regions of the mold. In addition, some researchers also investigated the influence of different mold materials selection on the temperature. Zhang et al. [10] used a CFD model to study the influence of two materials, copper and steel, on the temperature of large-framed molds. They found that using copper as the mold material can significantly improve the temperature uniformity of the mold. Zobeiry et al. [11] observed the thermal response of molds in the autoclave using the infrared thermography method and similarly found that the mold material and substructure are important parameters affecting the thermal distribution of the mold. Park [12] pointed out that the thermal response characteristics of the mold during the autoclave processing are significantly influenced by the mold material selection and the geometric features of the mold. In their study, a combined experimental and numerical approach was used to compare three identical geometric molds made of different materials: CFRP, aluminum, and invar. The study found that the thermal diffusivity of the mold plays a significant role in controlling the temperature distribution on the mold facesheet. In addition to mold geometric features and materials selection, the auxiliary materials and the reaction heat of the composite also affect the heat distribution of the mold during the curing process. Xie et al. [13] established a CFD model to analyze the mold temperature during the autoclave processing. Importantly, their model incorporates the influence of auxiliary materials. Weber et al. [14] suggested that enhancing the mold thermal performance in an autoclave can be achieved by reducing enclosed spaces beneath the auxiliary materials and eliminating fairings, as well as structures resembling “thermos flasks.” These adjustments have a notable positive impact on the heat transfer uniformity and efficiency during autoclave processing. Rasekh [15] found that the laminate thickness does indeed impact mold temperature. This thickness is not only linked to the amount of reaction heat but also influences the rate at which the heat is transferred to the mold.

While previous studies have investigated ways to enhance the thermal performance of molds, there is a lack of a quantitative analysis that considers the prioritization of these factors and the intricate nature of their interactions. The prioritization of factors will steer designers toward investing more time and effort in exploring the factors with higher priority during the mold design phase. Therefore, a quantitative analysis is required to prioritize the significance of these factors, examine their interactions, and comprehensively evaluate their collective impact on mold surface temperature.

This paper applies the Sobol method to conduct a detailed global sensitivity analysis (GSA), aiming to quantify the individual effects of each factor and their interactions with other factors on the variation in the mold surface temperature during the autoclave processing. In this method, the variance-based sensitivity indices provide a scalar measure of relative influence based on conditional variance, which is widely recommended by researchers [16,17,18,19,20] for effectively characterizing single- and multi-parameter interactive sensitivities in non-linear and non-additive models, and the relatively straight forward interpretation of the sensitivity indices. There are five factors considered in this analysis including CHTC, TCL, TMF, MMT, and TAL used in the autoclave processing. The analysis not only identifies the most significant and least influential factors among the above-mentioned, but it also reveals the impacts of their interactions on mold surface temperature. Furthermore, it investigates how different ranges of values for these factors affect their sensitivity. The findings of this study are expected to provide a thorough understanding of factors sensitivity and serve as a clear guideline for designers involved in mold design and associated heating equipment design.

## 2. Mathematical Model of Curing Process

### 2.1. Cure Kinetic Model

A cure kinetics model describes the curing reaction of the resin in composite material and is typically expressed by the following equation:

(1) $$\frac{d\alpha }{dt}=k(T)f(\alpha )$$

where *α* is the degree of cure, *T* is the temperature, *f*(*α*) is the mechanism function for the curing reaction, closely related to the resin type and curing conditions. According to the form of the mechanism function, cure kinetics can be divided into the following two categories:

(1) Nth-order Model

The Nth-order kinetic model, which is expressed as follows, has the significant feature of maximizing the reaction rate during the early phases of curing.

(2) $$\frac{d\alpha }{dt}=k(T){\left(1-\alpha \right)}^{n}$$

(2) Autocatalytic Model

The autocatalytic curing reaction exhibits an initial phase during which the degree of cure begins to increase. As the degree of cure continues to progress, the curing rate reaches its maximum and then gradually decreases, an expression commonly described as:

(3) $$\frac{d\alpha }{dt}=k(T){\left(1-\alpha \right)}^{n}\alpha^{m}$$

where *n* and *m* are the reaction order.

In the cure kinetic model, the relationship between the reaction rate and temperature is typically described by the Arrhenius equation. The *k*(*T*) can be expressed as follows:

(4) $$k(T)=Ae^{-\frac{E_{a}}{RT}}$$

where *A* is the pre-exponential factor, *E*_a_ is the activation energy for the reaction, and *R* is the gas constant.

### 2.2. Thermo-Chemical Model

This model provides a comprehensive understanding of the relationship between heat transfer and curing reaction. It enables predictions concerning the behavior of curing and the thermal response. The internal heat generated during the curing reaction is considered an integral heat source. By substituting this heat source Equation (6) into the Fourier heat conduction Equation (5), a thermos-chemical model can be derived.

(5) $$\frac{\partial (\rho_{c}C_{p}T)}{\partial t}=\frac{\partial }{\partial x_{i}}(k_{ij}\frac{\partial T}{\partial x_{j}})+{\dot{q}}_{0}\;(i,j=1,2,3)\;$$

(6) $${\dot{q}}_{0}=\rho_{r}V_{r}Q_{R}\frac{d\alpha }{dt}$$

where *ρ*_c_ is the density of composite, *C*_p_ is the specific heat capacity of composite, *k*_11_ is the thermal conductivity of composite in the fiber direction, *k*_22_, *k*_33_ are the thermal conductivity of the composite in the cross-fiber direction, *t* is the time, *ρ*_r_ is the density of the resin, *V*_r_ is the volume of the resin, *Q*_R_ is the heat reaction of the resin, ${\dot{q}}_{0}$ is the rate of heat release during resin curing reaction. The degree of cure is calculated by integrating the instantaneous rate of cure:

(7) $$\alpha =\int_{0}^{t}\frac{d\alpha }{dt}dt$$

## 3. Establishment and Validation of the Finite Element (FE) Model

### 3.1. Autoclave Processing and Materials

**Figure 1:**
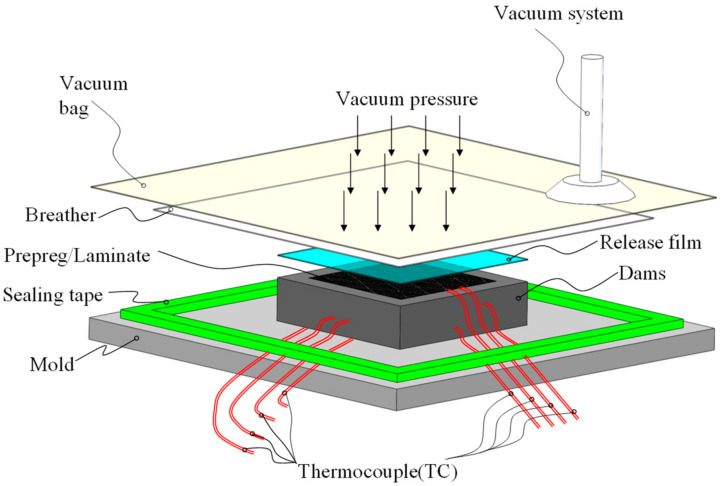
Preparation of composite laminates and mold for autoclave processing.

Autoclave processing is commonly used in the manufacturing of CFRP components for high-performance applications, especially for large and complex structures. This method involves cutting and layering thin sheets of high modulus fiber impregnated with partially cured resin (prepreg or laminate) to achieve the desired component shape. Once assembled, the structure is covered with multilevel auxiliary materials of cloth (bleeder and breather) and sealed within a vacuum bag (see Figure 1). The autoclave acts as a temperature and pressure-controlled vessel, with the vacuum system connected to the bag. To facilitate the curing process, pressure and temperature are applied to the structure according to a predetermined profile. The temperature profile initiates the resin polymerization reaction, while the pressure ensures that the prepreg properly conforms to the surface of a mold facesheet, achieves compaction at the desired fiber volume fraction, and prevents void formation during resin cure.

**Figure 2:**
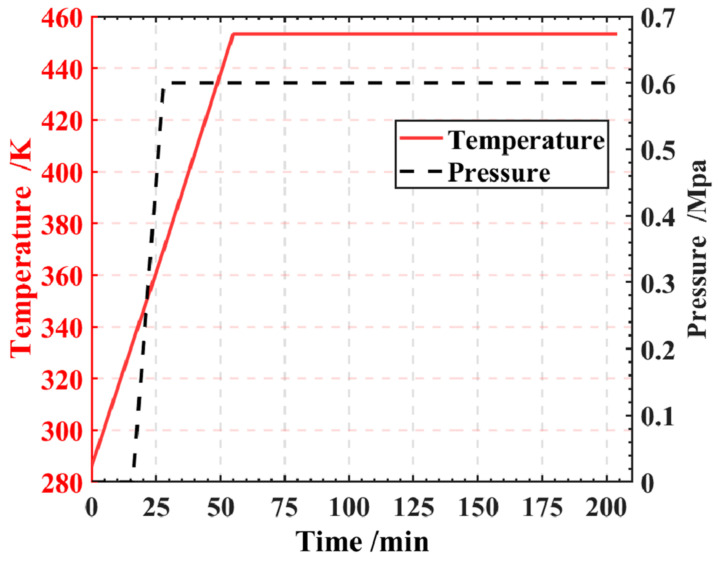
Temperature and pressure profiles of the curing process.

According to the process requirements provided by the prepreg supplier, the prepreg undergoes curing during the heating and holding stages, while minimal curing reaction occurs during the subsequent cooling stage, resulting in no reaction heat release at that stage. Therefore, we will only focus on the heating and holding stages. Figure 2 illustrates the temperature and pressure profiles for these stages. The temperature was heated from room temperature to 180 °C, with a heating rate of 3 °C/min, and then held for 150 min, and the pressure started to increase from 1 atm to 0.6 Mpa within 10 min when the temperature reached 60 °C in the autoclave and then maintained throughout the holding stages.

**Figure 3:**
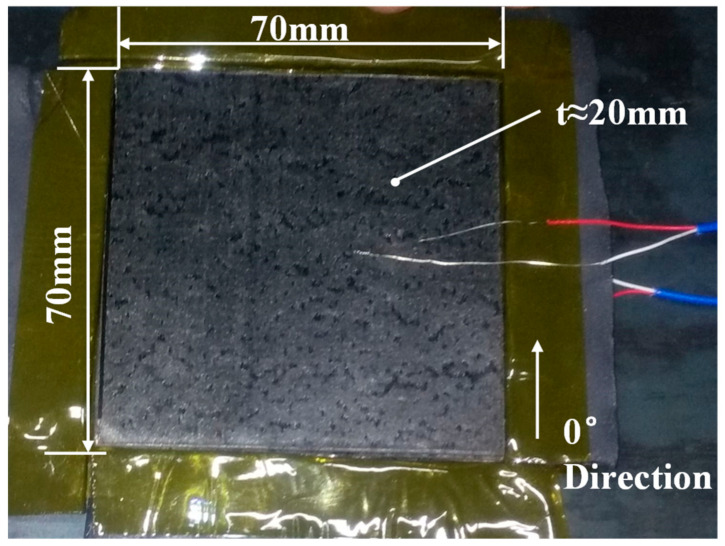
Schematic of composite laminates dimensions and layup orientation.

**Figure 4:**
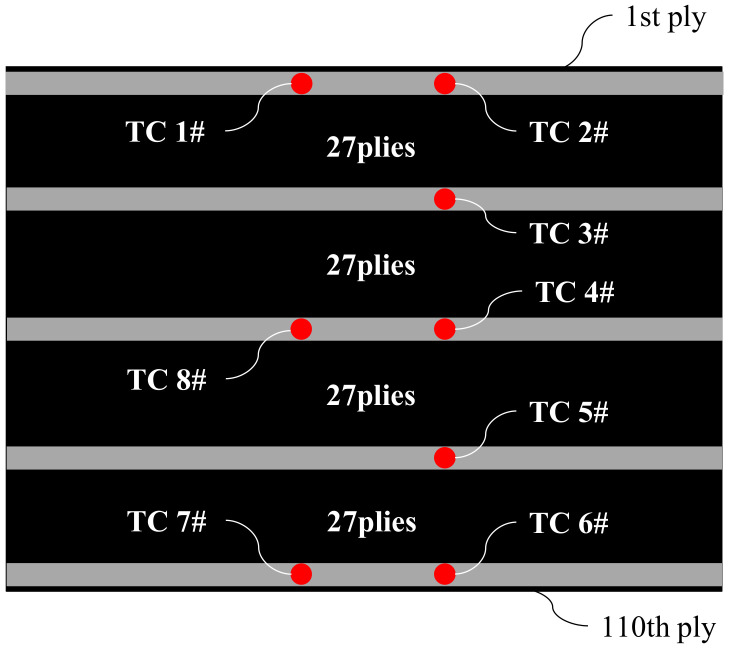
Schematic of thermocouples arrangement.

The experimental material, namely CYCOM X850^®^, is produced by Cytec Industries (Woodland Park, NJ, USA). It consists of toughened epoxy thermoplastics and unidirectional T800 fibers, with a fiber volume fraction of 65% and a density of 190 g/m^2^. The composite laminates have dimensions of 70 mm × 70 mm × 20 mm and are stacked on the mold in the 0° direction, as shown in Figure 3. Prior to stacking, the mold surface is coated with the release agent LOCTITE^®^ FREKOTE 770NC (Henkel, Dusseldorf, Germany). A total of 110 layers are stacked. The location of the thermocouples is shown in Figure 4. Table 1 and Table 2 display the thermal physical properties and cure kinetic constants for X850. The total heat released during curing is determined as *Q*_R_ = 119.2 J/g.

### 3.2. Establishment of the FE Model of Curing Process for the Composite Laminates

**Figure 5:**
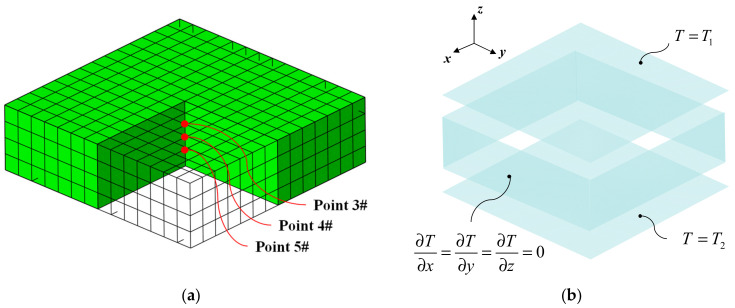
The FE model for curing process of the composite laminates: (**a**) description of mesh and temperature monitoring locations; (**b**) description of boundary condition.

The curing process of composite materials is simulated using ABAQUS 6.13-1, a commercial finite element software, along with FORTRAN subroutines: HETVAL, USDFLD, DISP, and FILM. HETVAL defines the reaction heat within the composite; USDFLD delineates the curing degree field; DISP sets the temperature boundary conditions; and FILM establishes the convective heat transfer boundary conditions. HETVAL and USDFLD are crucial subroutines for describing the composite laminate’s thermal–chemical curing model. The FE model with the 20-node quadratic heat transfer brick (DC8D20), was illustrated in Figure 5. This model comprises 784 elements and 4125 nodes, as shown in Figure 5a. This model is used to validate the accuracy of composite material parameters shown in Table 1 and Table 2, excluding other influencing factors. In order to consider the impact of other factors on the mold surface temperature, an auxiliary materials–laminates–mold FE model will be established in Section 3.4. To assess the model’s accuracy, temperature monitoring points at Point 3, Point 4, and Point 5 were compared with the measurements taken by thermocouples TC3#, TC4#, and TC5# as shown in Figure 4.

**Figure 6:**
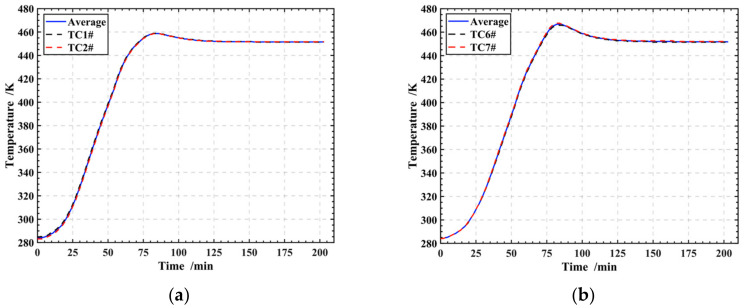
Temperature boundary conditions of the FE model for the laminates curing process: (**a**) *T*_1_; (**b**) *T*_2_.

The thermal boundary conditions in the cure simulation FE model are displayed in Figure 5b. To ensure the consistency of boundary conditions and eliminate deviations caused by differences between the experiment and the simulation, the prescribed temperature boundary condition *T*_1_ was applied to the upper surface of the laminates, which was determined from the average temperature recorded by thermocouples TC1# and TC2# (as shown in Figure 4). Similarly, *T*_2_ was applied to the bottom surface using the average temperature recorded by thermocouples TC6# and TC7#. For the remaining four sides of the laminates, adiabatic boundary conditions were applied, as depicted in Figure 5b. Furthermore, Figure 6 illustrates the temperature and average values collected by thermocouples TC1#, TC2#, TC6#, and TC7#.

### 3.3. Validation of the FE Model of Curing Process for the Composite Laminates

**Figure 7:**
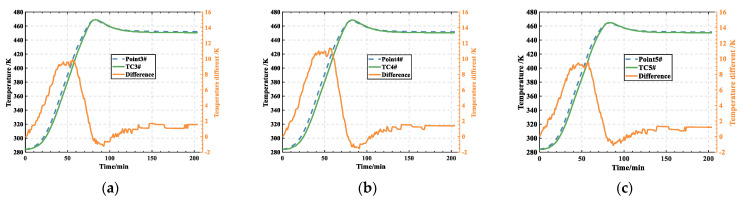
Comparison results and deviation between experiment and simulation: (**a**) 3#; (**b**) 4#; (**c**) 5#.

Temperatures at Point3#, Point4#, and Point5#, as depicted in Figure 5a, were compared with those recorded by thermocouples TC3#, TC4#, and TC5# (as shown in Figure 4) during the experiment. The results of this comparison show a maximum deviation of 11.4 K, corresponding to a relative error of 8.8% (as shown in Figure 7). This slight deviation can be attributed to a modeling assumption that the laminates’ surroundings are adiabatic, while heat exchange occurs during the curing process. As a result, the simulated temperatures consistently exceed the measurements taken by the thermocouples. The comparison shows that the simulation model is reliable and is capable of accurately predicting the thermal behavior of the laminates during cure.

### 3.4. Establishment of the FE Model of Curing Process for Auxiliary Materials-Laminates-Mold

**Figure 8:**
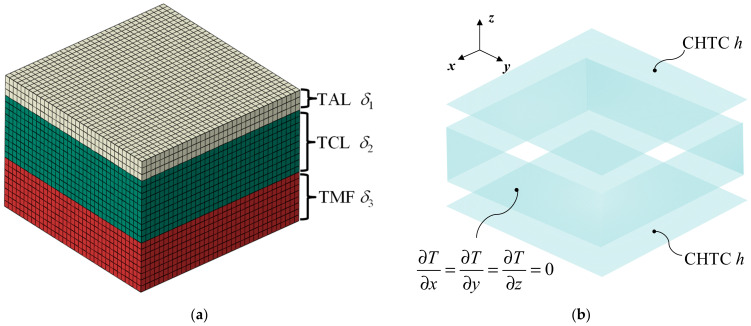
The FE model of curing process for auxiliary materials-laminates-mold: (**a**) description of mesh and layers thicknesses; (**b**) description of boundary condition.

To analyze the other factors that influence the temperature of the mold surface, it is necessary to establish an FE model (as shown in Figure 8) for the curing process. This model includes the following factors: the airflow state and velocity distribution in the autoclave, the composite laminates, the shape of the mold, the thermal physical properties of the mold material, and the auxiliary materials consisting of the vacuum bag, porous Teflon, breath cloth and bleeder. To simplify the model and improve calculation efficiency, the auxiliary materials are combined into an effective layer with a thickness of *δ*_1_ and covering the composite laminates (with thickness *δ*_2_) and the mold facesheet (with thickness *δ*_3_). The upper and bottom surfaces are set as the Robin boundary conditions, while the remaining four surfaces are set as adiabatic boundary conditions shown in Figure 8b. The reasons for setting Robin boundary conditions and the ranges of CHTC, thicknesses, and other parameters will be discussed in Section 4.2. Additionally, the thermal resistance at the interfaces between the three regions was assumed to be zero.

## 4. Sensitivity Analysis Theory

### 4.1. Sobol’s Method

Sobol’s method is a GSA that uses variance analysis to assess the impact of different factors on the model output. It decomposes the output variance into individual input factor variances and their interactions, allowing for the calculation of sensitivity indices for each input factor. These indices provide a means to evaluate the contributions of various factors to the model output.

#### 4.1.1. Sobol’s Sensitivity Indices

In the variance-based sensitivity analysis, any model can be seen as a function *Y* = *f*(***Z***), where ***Z*** is a d-dimensional vector consisting of model input variables (*Z*_1_, *Z*_2_, … *Z*_d_), and *Y* is the specified univariate model output. Sobol [23] proved that *f*(***Z***) has the following decomposition:

(8) $$Y=f_{0}+\sum_{i=1}^{d}f_{i}(Z_{i})+\sum_{i<j}^{d}f_{ij}(Z_{i},Z_{j})+\cdots +f_{1,2,\ldots ,d}(Z_{1},Z_{2},\ldots ,Z_{d})$$

where *f*_0_ is a constant. The decomposition of the model output variance Var(*Y*) is given by:

(9) $$\mathrm{Var}(Y)=\sum_{i=1}^{d}V_{i}+\sum_{i<j}^{d}V_{ij}+\cdots +V_{12\ldots d}$$

where

(10) $$\mathrm{Var}(Y)=\int_{0}^{1}f^{2}(Z)dZ-f_{0}^{2}=\sum_{s=1}^{d}\sum_{i_{1}<\ldots <i_{s}}^{d}\int f_{i_{1}\ldots i_{s}}^{2}dZ_{i_{1}}\ldots dZ_{i_{s}}$$

(11) $$V_{i}=Var_{Z_{i}}(E_{Z_{\sim i}}(Y\left|Z_{i}\right.))$$

(12) $$V_{ij}={\mathrm{Var}}_{Z_{ij}}(E_{Z_{\sim ij}}(Y\left|Z_{i}\right.,Z_{j}))-V_{i}-V_{j}$$

Using analogous reasoning, we can obtain *V*_ijk_, …, *V*_123…*d*_ according to Equations (10)–(12), where ***Z***_~*i*_ = (*Z*_1_, *Z*_2_, …, *Z_i_*_−1_, *Z_i_*_+1_, …, *Z_d_*) represents all input random variables except Zi remaining unchanged. The notation Var (·) and E (·) is used to represent the variance and expectation operations, respectively. To derive the following equation, both sides of Equation (9) are divided by Var(*Y*):

(13) $$1=\sum_{i=1}^{d}S_{i}+\sum_{i=1,j\neq i}^{d}S_{ij}+\ldots +\sum_{i=1,j\neq i,\;k\neq j}^{d}S_{ij\ldots k}$$

where *S_i_* are the first-order sensitivity indices, *S_ij_* are the second-order sensitivity indices, *S*_12…*d*_ are the high-order sensitivity indices. These indices can be used to quantify the importance of each input variable in influencing the model output.

The expression for the total effect sensitivity indices can be derived from the above, and it is as follows:

(14) $$S_{Ti}=\frac{E_{Z_{\sim i}}({\mathrm{Var}}_{Z_{i}}(Y\left|Z_{\sim i}\right.))}{\mathrm{Var}(Y)}=1-\frac{{\mathrm{Var}}_{Z_{\sim i}}(E_{Z_{i}}(Y\left|Z_{\sim i}\right.))}{\mathrm{Var}(Y)}$$

*S*_T*i*_ can be used to quantify the contribution of input variable *Z_i_* to the uncertainty in the model output. This contribution includes the uncertainty in the model output caused by any sequential interactions between *Z_i_* and any other input variables.

#### 4.1.2. Definition of the Output Variable

**Figure 9:**
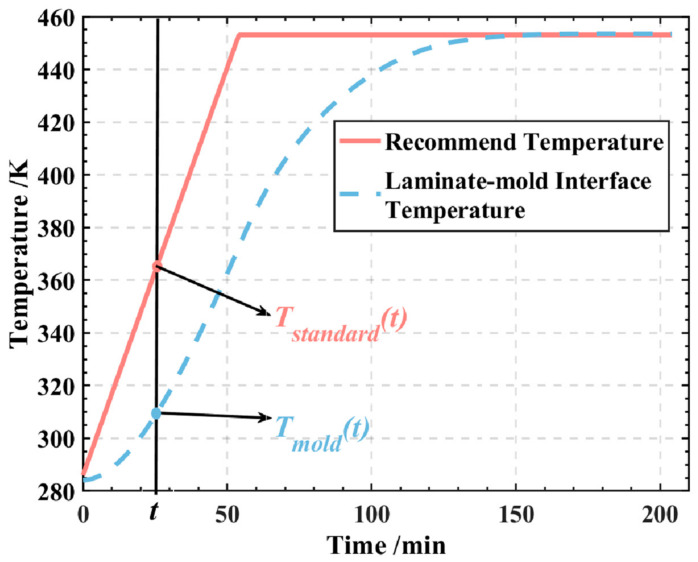
Schematic diagram of the output variable *Y*.

In this analysis, the temperature at the laminate–mold interface is of primary concern, as the temperature distribution on the mold surface plays a critical role in enhancing the curing quality. The output variable, denoted as *Y*, is defined according to Figure 9, and can be expressed using the following formula:

(15) $$Y=\int_{0}^{t_{D}}\sqrt{{\left[T_{\mathrm{standard}}(t)-T_{\mathrm{mold}}(t)\right]}^{2}}dt$$

where the duration of the heating stage and the holding stage is represented by *t*_D_. At any given time, *T*_standard_(*t*) indicates the temperature in the recommended temperature profile, while *T*_mold_(*t*) represents the simulated temperature at the interface between the laminates and the mold.

### 4.2. Selection of Parameters and Ranges

Parameter selection is essential to quantify the factors affecting mold surface temperature. In this context, factors such as airflow state, velocity distribution within the autoclave, dimensions of composite components or structures, composite materials, mold structure construction, mold materials, and auxiliary materials are considered.

1. Autoclave Heat Transfer and CHTC: Influencing Factors and Analysis

In the autoclave environment, heat transfer from the turbulent gas (typically air or nitrogen) into the mold primarily occurs through forced convection, where the effectiveness is summarized by CHTC. When molds are loaded into the autoclave, the airflow underneath them becomes disturbed, resulting in highly complex and non-uniform turbulent gas flow patterns. This disturbance leads to an uneven distribution of CHTC around the mold, which results in an uneven distribution of heat flux on the mold surface, leading to variations in temperature across the mold surface. Conversely, a uniform distribution of CHTC around the mold ensures more consistent heating throughout the autoclave. Therefore, CHTC distribution is crucial for mold surface temperatures, which in turn directly influence the quality and properties of the CFRP components being manufactured. CHTC is influenced by various factors [24], including flow medium, flow velocity, pressure, mold geometry, substructure configurations, location in the autoclave, and mold nesting and loading scenarios. It can be used as the correct characterization of factors concerning heat transfer and boundary conditions. Generally, CHTC for natural convection and forced convection of air fall within the ranges of 5–25 W/m^2^/K and 10–100 W/m^2^/K, respectively. Considering the pressure in the autoclave, the range of CHTC variation can be set between 10 and 300 W/m^2^/K [25,26].

2. Curing Reaction and Heat Release in Composite Laminates

The curing reaction depends on temperature history, resin types, and curing agents. Even when using the same resin types and curing agents, laminates with different thicknesses exhibit varied temperature histories, as demonstrated by Yi et al. [27]. In situations involving complex-shaped composite components or structures with varying laminate thicknesses, the thickness of the composite laminates can be considered as an input parameter for the sensitivity analysis. In this sensitivity analysis, the study focuses on analyzing thickness variations ranging from 1 mm to 20 mm.

3. Frame Mold Configuration and Material Selection

In autoclave processing, a commonly employed mold is the frame mold, consisting primarily of facesheet and substructure [28]. The facesheet thickness should be considered as an input parameter for sensitivity analysis, with a range typically set from 8 to 25 mm. The impact of the substructure on the airflow state and velocity distribution can be characterized by the CHTC, as discussed in the previous section. In addition to the substructure and facesheet thickness, the thermal properties of the mold material, particularly diffusivity, significantly affect the mold’s heating characteristics [12]. Thus, material selection becomes an input parameter for sensitivity analysis. Table 3 displays the thermal physical properties parameters, including density, specific heat, and thermal conductivity, for seven commonly used mold materials.

4. Auxiliary Materials

The autoclave processing utilizes various auxiliary materials, including bleeder, breath, sealing, and vacuum bags. To streamline the model, these materials are treated collectively as an effective layer termed the auxiliary material layer. The upper auxiliary materials layer is thicker, providing substantial resistance to heat transfer. A thickness increase in the auxiliary materials layer is anticipated to notably change the temperature distribution in laminates and the mold facesheet. Generally, the auxiliary layer has a thickness ranging from 1 mm to 7 mm. Table 3 lists the thermal physical property parameters of this auxiliary layer.

The selected parameters along with their respective default ranges are displayed in Table 4.

## 5. Results and Discussion

### 5.1. Sensitivity Indices of Parameters

**Figure 10:**
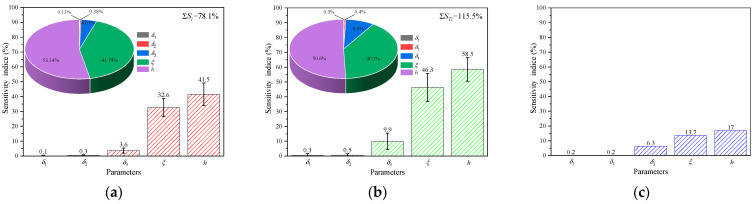
Sensitivity indices, with 95% confidence bounds, and for parameters within the default ranges: (**a**) first-order sensitivity indices; (**b**) total-order sensitivity indices; (**c**) interaction sensitivity indices.

In order to ensure the stability of sensitivity indices, this sensitivity analysis generates a sample of size *N* = 2000 for each parameter, utilizing the default parameter ranges as outlined in Table 4. The results of this analysis, obtained using Sobol’s method with the default parameter ranges, are presented in Figure 10. Notably, the sensitivity indices exhibit significant variations. From the pie charts, the various contributions of each parameter to the mold surface temperature are accounted for by the clear differences in the sensitivity indices of the parameters. In Figure 10, it can be clearly observed that the parameter CHTC is the most important parameter, and the parameters MMT and TMF are the second and third most influential parameters, respectively. It has shown that the thermal performance of the mold is significantly influenced by CHTC. Mold designers should focus on optimizing the geometric parameters of substructures to enhance efficiency. Since the substructure obstructs the airflow under the facesheet, it results in changes to the CHTC and heat flux. Aside from affecting the airflow pattern, the substructure also increases the surface area of mold exposed to heat, increasing heat flux to the mold, and adding thermal mass to the mold, thus decreasing heat flux to the mold. It is these opposing mechanisms that contribute to the rational distributions of hot and cold spots on the mold surface by optimizing the substructure. According to the pie charts in Figure 10, the sums of the first-order indices and total-order indices for the three parameters account for 99.5% and 99.3% of that of all parameters. Therefore, the remaining parameters exert either negligible or minor effects on the mold surface temperature. These findings are consistent with the conclusions of previous studies by Xie et al. [29] and Huang et al. [30], suggesting that heat release during the curing process and the thickness of the auxiliary material layer have negligible effects on the mold surface temperature. Therefore, it is sufficient to consider only the thermal response of molds in the design phase. The first-order sensitivity indices of TAL, TCL, TMF, MMT, and CHTC are 0.1%, 0.3%, 3.6%, 32.6%, and 41.5%, respectively. The total-order sensitivity indices of TAL, TCL, TMF, MMT, and CHTC are 0.3%, 0.5%, 9.9%, 46.3%, and 58.5%, respectively. The total-order sensitivity index values are higher than that of the first-order sensitivity index, which can reveal the impacts of parameter interactions since the total-order sensitivity index contains both first-order and interaction effects. Furthermore, the sum of first-order sensitivity indices (∑*S_i_*) is 78.1%, which is not equal to 1, and the difference 1 − ∑*S_i_* reflects the presence of interactions in the model. This suggests that the individual parameter (78.1%) has a significantly higher influence on mold surface temperature than the interactions (21.9%) for the five parameters. It is prudent to focus major efforts on individual parameters in optimizing molds.

**Figure 11:**
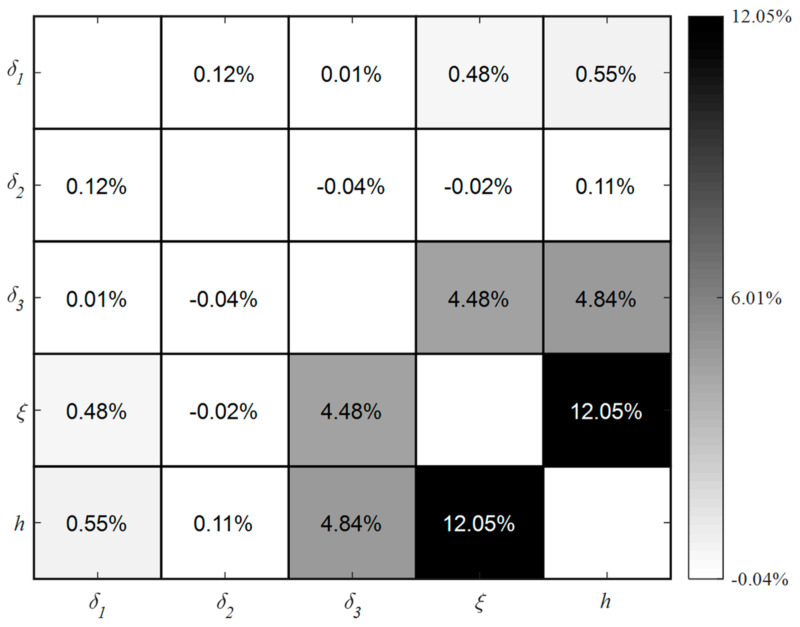
Second-order sensitivity indices between five parameters within the default ranges.

Among all parameters, the total interaction effects amount to 21.9%. The primary interaction sources affecting mold surface temperature are the interactions between CHTC and the other four input factors, which account for 17% as depicted in Figure 10c, constituting 77.6% of the total interaction effects. Second-order indices are necessary for a detailed understanding of interactions between different input factors, illustrated in Figure 11. Dark grey shading indicates high parameter interactions, while light grey shading signifies low parameter interactions. The maximum value of the second-order indices is attributed to the pair (CHTC, MMT), which are the two significant input factors that cannot be overlooked in the variation in mold surface temperature. In addition, the interaction in the pair between CHTC and the other two input factors (MMT and TMF) is over 4.48%, which is far larger than the interaction in other pairs. As a result, for the three input factors, CHTC, MMT, and TMF, it is possible to assume that the effect of one element may rise as the other two parameters vary, as well as the uncertainty produced by the other two parameters, which may be more or less relevant. Furthermore, the sum of first-order and second-order indices for CHTC, namely 41.5% (Figure 10) and 17.6% (Figure 11), is 59.1% falling within the 95% confidence interval of the total-order index of 58.5%. Similar results are observed for the parameters MMT and TMF. This means that for the variance of mold surface temperature, the three parameters, CHTC, MMT, and TMF, contribute mostly to direct effects and second-order interactions, and little to higher-order interactions (3-order, 4-order, etc.). Therefore, the interaction effects between all factors resulting from high-order interactions (excluding second-order interaction effects) can be ignored, which helps reduce the complexity of thermal optimization for the mold. The presence of negative signs (−0.04% and −0.02%) can be attributed to numerical errors in the estimations. Such negative values are commonly encountered in Saltelli’s Monte Carlo simulations (MCS) when the sensitivity indices approach zero [31].

### 5.2. Impacts of Parameter Variation Range on Sensitivity Indices

Sensitivity index variations can result from parameter range variations [32,33]. Thus, it is essential to investigate the effects of various ranges on the sensitivity indices. In this section, first, the TAL ranges are broadened to 1–13 mm, 1–19 mm, and 1–25 mm, the TCL range are broadened to 1–39 mm, 1–58 mm, and 1–77 mm, and the remaining are consistent with Table 4. Subsequently, only the CHTC ranges are set to 10–155 W/m^2^/K and 155–300 W/m^2^/K, and the others are consistent with Table 4.

#### 5.2.1. Influence of TAL and TCL Range on Sensitivity Indices

**Figure 12:**
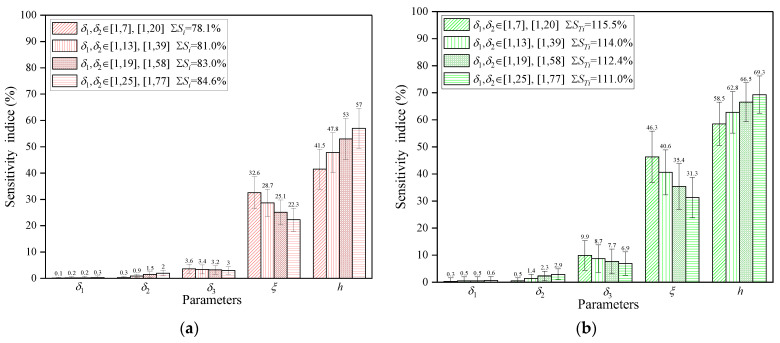
Sensitivity indices of parameters with 95% confidence bounds: (**a**) first-order sensitivity indices for different ranges of TAL and TCL; (**b**) total-order sensitivity indices for different ranges of TAL and TCL.

Figure 12 presents sensitivity analysis results for different ranges of TAL and TCL. These results indicate that the first-order and total-order sensitivity indices for each parameter vary slightly, but the sensitivity rankings of the parameters remain the same within different TAL and TCL ranges. As illustrated in Figure 12, wider ranges for TAL and TCL result in higher sensitivity indices compared to the default range. Furthermore, expanding the TAL and TCL ranges results in a modest decrease in sensitivity to TMF and MMT and an increase in sensitivity to CHTC. In addition, it also leads to a small increase in the portion of the variation in mold surface temperature contributed by the effect of the individual parameter and a slight decrease in the portion contributed by the interaction.

**Figure 13:**
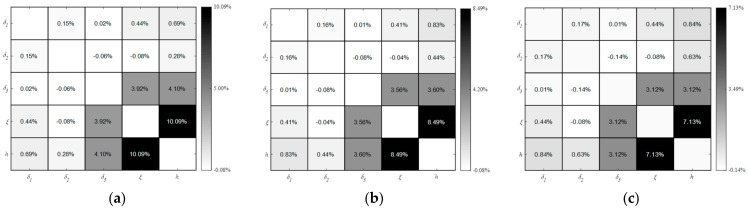
Second-order sensitivity indices between five parameters based on different ranges of TAL and TCL: (**a**) *δ*_1_ ∈ [1, 13], *δ*_2_ ∈ [1, 39]; (**b**) *δ*_1_ ∈ [1, 19], *δ*_2_ ∈ [1, 58]; (**c**) *δ*_1_ ∈ [1, 25], *δ*_2_ ∈ [1, 77].

The results of the second-order sensitivity indices for different ranges of TAL and TCL are displayed in Figure 13, which indicate modest differences in the second-order sensitivity indices. A comparison of Figure 11 and Figure 13, shows that the increase in the wide TAL and TCL ranges gives rise to a negligible increase or decrease in the second-order sensitivity indices of all parameters, but the parameters CHTC and MMT still exhibit the most significant second-order effects. The above investigations suggest that the sensitivity indices are frequently dependent less on the ranges of TAL and TCL. Based on the variation trend of sensitivity indices for different ranges of TAL and TCL, it can be inferred that the effect of composite laminates and auxiliary materials on the mold surface temperature should be regardless of the mold design phase.

#### 5.2.2. Influence of CHTC Range on Sensitivity Indices

**Figure 14:**
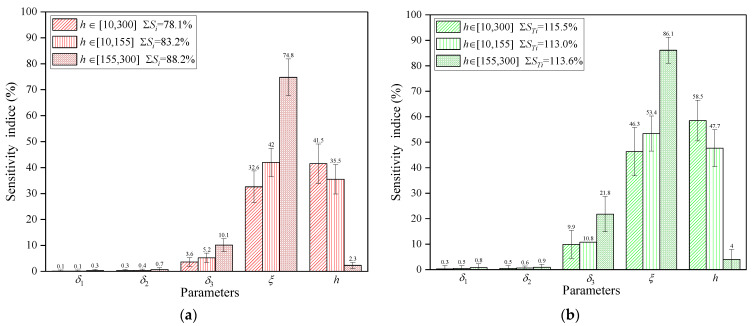
Sensitivity indices of parameters with 95% confidence bounds: (**a**) first-order sensitivity indices for different ranges of *h*; (**b**) total-order sensitivity indices for different ranges of *h*.

Figure 14 illustrates the sensitivity indices for various CHTC ranges, revealing notable fluctuations in sensitivity and parameter rankings. When the CHTC range is split into two intervals of equal width but covering different values, the first-order and total-order sensitivity indices decrease for CHTC while increasing for other parameters, as opposed to the default range. Consequently, MMT emerges as the most sensitive parameter. Particularly noteworthy is the sharp decline in sensitivity indices for the CHTC parameter when it falls within the range of 155–300 W/m^2^/K, rendering its impact largely negligible. These findings demonstrate the substantial impact of the CHTC range on both sensitivity indices and their relative significance. In the autoclave, a convective heating system, the distribution of CHTC exhibits significant spatial variations, and the airflow pattern and velocity around the mold generate regions with both high and low CHTC across the mold surface. An effective mold substructure design should enhance flow velocity under the mold facesheet, thereby improving the CHTC range by narrowing it down and raising the lower limit. Optimizing the mold substructure design before selecting the mold material is a prudent approach. It is futile to attempt to enhance thermal performance solely by deciding on the mold material without regulating the CHTC range across the mold surface through an optimized mold substructure.

**Figure 15:**
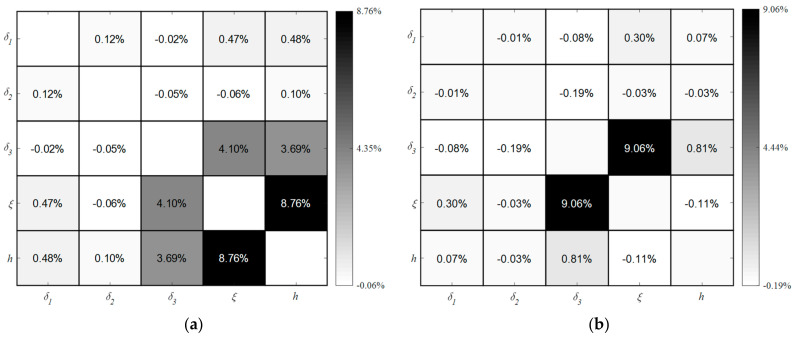
Second-order sensitivity indices between five parameters based on different ranges of CHTC: (**a**) *h* ∈ [10, 155]; (**b**) *h* ∈ [155, 300].

Figure 15 provides a detailed representation of second-order indices, which quantifies the impact of interactions between two parameters on the temperature variation on the mold surface within two distinct CHTC ranges, namely 10–155 W/m^2^/K and 155–300 W/m^2^/K. A comparison of Figure 11 and Figure 15a, reveals a marginal change in the second-order sensitivity indices of all parameters. The primary contributors to these interactions are three pairwise relationships: CHTC vs. MMT, CHTC vs. TMF, and MMT vs. TMF. Notably, the parameters CHTC and MMT maintain the most significant second-order influence when CHTC falls within two different ranges, namely 10–155 W/m^2^/K and 10–300 W/m^2^/K. Comparing Figure 11 and Figure 15a shows that the CHTC range has a great influence on three pairwise interactions: CHTC vs. MMT, CHTC vs. TMF, and MMT vs. TMF.

The investigations above reveal that the ranges of TAL and TCL have a minimal impact on the sensitivity indices and their relative importance. However, the CHTC range significantly influences these indices. Additionally, as depicted in Figure 12 and Figure 14, for the different ranges of CHTC, TAL, and TCL, the first-order sensitivity indices of CHTC, MMT, and TMF contribute to over 97.3% of the total sum of first-order sensitivity indices for all parameters. Consequently, these three parameters, CHTC, MMT, and TMF, play a crucial role in influencing mold surface temperature and are pivotal considerations during the design phase for optimizing molds.

## 6. Conclusions

In this study, GSA has been performed on a thermal–chemical curing FE model based on Sobol’s method, and the difference between the mold surface temperature and the standard process temperature was used as the response quantity of interest. The sensitivity indices of all parameters in the model were systematically investigated. The main conclusions can be obtained as follows:

(1) The sensitivity order of the temperature of the mold surface for the five parameters is CHTC > MMT > TMF > TCL > TAL. In addition, the mold surface temperature is mostly influenced by CHTC, MMT, and TMF. The other parameters, TCL and TAL, have negligible or minor influence on the mold surface temperature and the thermal response is dominated by the mold.
(2) The results of the sensitivity indices for different ranges of TAL and TCL infer a negligible effect on the sensitivity indices and rankings of the parameters. However, the sensitivity indices and rankings of the parameters are significantly dependent on the different ranges of CHTC. Although variations in the ranges of parameters can affect the sensitivity indices and rankings, but cannot change the fact that CHTC, MMT, and TMF play a decisive role in influencing mold surface temperature. The reason for this is that the sum of first-order sensitivity indices of these three parameters accounts for over 97.3%.
(3) Based on an understanding of the impact of various CHTC ranges on sensitivity indices, a mold design strategy can be outlined: it is advisable to prioritize optimizing the mold substructure design before finalizing the mold material.
(4) The analysis reveals the individual effects of each parameter and its interactions with other parameters. The individual effects of each parameter contribute to a significant portion (78.1%) of the variation in mold surface temperature. This implies that the individual effect of each parameter has a more important effect on mold surface temperature, while the effects of interactions among parameters on mold surface temperature are low and can be ignored. Therefore, when optimizing and regulating the uniformity of mold surface temperature distribution, it is crucial to focus on adjusting and optimizing the individual parameters.

The current study is limited as only one type of composite material was considered. Future research will explore the impact of employing various composite materials with distinct thermos-chemical models. Additionally, this paper did not delve into the correlation between mold substructure variation and the distribution of CHTC, which warrants further investigation.

## Figures and Tables

**Table 1:** The cure kinetic constants for X850 [21].

Materials	*A*/(1/s)	*E* (J/mol)	*m*	*n*	*Q*_R_ (J/g)
X850	67,342.89	67,198.43	0.2857	1.2373	124.89
					132.12
					106.07
					106.63
					126.36

**Table 2:** Thermal physical properties parameters of X850 [22].

Parameters	Value
*C*_p_/(J/(kg·K))	−1.082 + 0.00502*T* − 0.0956*α* − 2.422 × 10^−6^ *T*^2^ − 0.0161*α*^2^
*k*_11_/(W/(m·K))	−1.875 + 0.00955*T* − 0.232*α* − 5.672 × 10^−6^ *T*^2^ − 0.0725*α*^2^
*k*_22_, *k*_33_/(W/(m·K))	−0.3 + 0.00141*T* + 0.0381*α* + 3.588 × 10^−6^ *T*^2^ − 0.354*α*^2^
*ρ*_c_/(kg/m^3^)	1.57 × 10^3^

**Table 3:** Thermal physical properties of the mold materials and the auxiliary material layer [28].

Materials	Thermal Conductivity /(W/m/K)	Density /(10^3^ kg/m^3^)	Specific Heat /(J/kg/K)	Code
Graphite	57.7	1.78	1046.7	1
Aluminum	201.2	2.7	963	2
Steel	50.5	7.86	460.5	3
Nickel	72.1	8.9	460.5	4
Carbon Fiber/Epoxy	3.46–6.06	1.5	1046.7	5
Glass Fiber/Epoxy	3.17–4.33	1.9	1256	6
Ceramic	1.44–11.54	2.75	3516.9–6280.2	7
Auxiliary Materials	0.12	0.88	802	/

**Table 4:** List of the parameters and their default ranges used for sensitivity analysis.

Parameter’s Number	Parameter’s Name	Parameter’s Notation	Unit	Range	Type
1	TAL	*δ*_1_	mm	[1, 7]	Continuous
2	TCL	*δ*_2_	mm	[1, 20]	Continuous
3	TMF	*δ*_3_	mm	[8, 25]	Continuous
4	MMT	*ξ*	/	1, …, 7	Discrete
5	CHTC	*h*	W/m^2^/K	[10, 300]	Continuous

## Data Availability

Data are contained within the article.
